# Evaluation of [^68^Ga]Ga-DOTA-TCTP-1 for the Detection of Metalloproteinase 2/9 Expression in Mouse Atherosclerotic Plaques

**DOI:** 10.3390/molecules23123168

**Published:** 2018-12-01

**Authors:** Max Kiugel, Sanna Hellberg, Meeri Käkelä, Heidi Liljenbäck, Tiina Saanijoki, Xiang-Guo Li, Johanna Tuomela, Juhani Knuuti, Antti Saraste, Anne Roivainen

**Affiliations:** 1 Turku PET Centre, University of Turku, FI-20520 Turku, Finland; max.kiugel@utu.fi (M.K.); sanna.hellberg@ki.se (S.H.); meeri.kakela@utu.fi (M.K.); halilj@utu.fi (H.L.); tiina.saanijoki@utu.fi (T.S.); juhani.knuuti@utu.fi (J.K.); antti.saraste@utu.fi (A.S.); 2 Turku Center for Disease Modeling, University of Turku, FI-20520 Turku, Finland; 3 Turku PET Centre, Åbo Akademi University, FI-20520 Turku, Finland; xiali@utu.fi; 4 Department of Cell Biology and Anatomy, University of Turku, FI-20520 Turku, Finland; johanna.tuomela@utu.fi; 5 Turku PET Centre, Turku University Hospital, FI-20520 Turku, Finland; 6 Heart Center, Turku University Hospital, FI-20520 Turku, Finland; 7 Institute of Clinical Medicine, University of Turku, FI-20520 Turku, Finland

**Keywords:** atherosclerosis, imaging, matrix metalloproteinase, positron emission tomography, plaque

## Abstract

*Background*: The expression of matrix metalloproteinases 2/9 (MMP-2/9) has been implicated in arterial remodeling and inflammation in atherosclerosis. We evaluated a gallium-68 labeled peptide for the detection of MMP-2/9 in atherosclerotic mouse aorta. *Methods*: We studied sixteen low-density lipoprotein receptor deficient mice (LDLR^-/-^ApoB^100/100^) kept on a Western-type diet. Distribution of intravenously-injected MMP-2/9-targeting peptide, [^68^Ga]Ga-DOTA-TCTP-1, was studied by combined positron emission tomography (PET) and contrast-enhanced computed tomography (CT). At 60 min post-injection, aortas were cut into cryosections for autoradiography analysis of tracer uptake, histology, and immunohistochemistry. Zymography was used to assess MMP-2/9 activation and pre-treatment with MMP-2/9 inhibitor to assess the specificity of tracer uptake. *Results*: Tracer uptake was not visible by in vivo PET/CT in the atherosclerotic aorta, but ex vivo autoradiography revealed 1.8 ± 0.34 times higher tracer uptake in atherosclerotic plaques than in normal vessel wall (*p* = 0.0029). Tracer uptake in plaques correlated strongly with the quantity of Mac-3-positive macrophages (R = 0.91, *p* < 0.001), but weakly with MMP-9 staining (R = 0.40, *p* = 0.099). Zymography showed MMP-2 activation in the aorta, and pre-treatment with MMP-2/9 inhibitor decreased tracer uptake by 55% (*p* = 0.0020). *Conclusions*: The MMP-2/9-targeting [^68^Ga]Ga-DOTA-TCTP-1 shows specific uptake in inflamed atherosclerotic lesions; however, a low target-to-background ratio precluded in vivo vascular imaging. Our results suggest, that the affinity of gelatinase imaging probes should be steered towards activated MMP-2, to reduce the interference of circulating enzymes on the target visualization in vivo.

## 1. Introduction

Atherosclerosis remains the leading cause of death in developed countries. Rupture of an atherosclerotic plaque often precedes complications of atherosclerosis, such as myocardial infarction or stroke. Plaques that are vulnerable to rupture are characterized by distinct morphological features, such as a large plaque and necrotic core volumes, positive vascular remodeling, and thin fibrous caps. On a cellular level, these plaques are characterized by active inflammation [1]. Targeted molecular imaging approaches have been tested for inflammation detection in atherosclerotic plaques, including [^18^F]FDG (a marker of glucose consumption by activated macrophages) [2]. Although [^18^F]FDG has been shown to characterize inflammation in atherosclerosis, more specific targets have been identified, such as the activation of matrix metalloproteinases in the vascular wall [3]. Peptide-based probes designed to detect specific target molecules can be conjugated with metal chelators. Thus, they have the advantage of easy and fast radiolabeling with a generator-produced radionuclide, excluding the necessity of an on-site cyclotron.

Gelatinases (matrix metalloproteinases 2 and 9 [MMP-2 and -9]) play a role in atherosclerosis. Gelatinases are secreted by several vascular cell types, including endothelial cells, pericytes, fibroblasts and myofibroblasts, macrophages derived from circulating monocytes, and local tissue macrophages [4,5], especially after differentiation [6,7]. Gelatinase activation may contribute to plaque rupture by destruction of the extracellular matrix (ECM) [8] or by promoting the death of macrophages [6,8] and smooth muscle cells [8]. Furthermore, MMP-9 has been associated with intraplaque hemorrhage in advanced atherosclerotic plaques [9]. Matrix metalloproteinases also contribute to the controlled ECM remodeling that is essential for migration and proliferation of smooth muscle cells, and is important in fibrous cap thickening and plaque stability. These apparently opposing actions prevent the therapeutic use of broad spectrum matrix metalloproteinase-targeting molecules or inhibitors [10,11]. Selective MMP inhibitor molecules, however, are being designed for the treatment of cardiovascular diseases [6].

In clinical trials, MMP-9 activity has been reported to be associated with the vulnerability of carotid artery plaques. Atherosclerotic plaque instability has been visualized by ultrasound as a markedly irregular or ulcerated surface, or as hypodense or heterogeneous structure [12]. Plaque vulnerability was also detected retrospectively, in the form of a thin fibrous cap and/or recent intraplaque hemorrhage on histology [13], as well as being indicated by a previous stroke or peripheral vascular disease [13]. MMP-9 does not only affect the thinning of the fibrotic cap around the lipid core, but it also contributes strongly to the overall atherosclerotic process [14]. A higher quantity of circulating MMP-9 in patients with coronary artery disease (CAD) seems to predict cardiovascular mortality [12,14]. Elevated circulating levels of MMP-2/9 are also strongly associated with the development of an acute myocardial infarction, rather than stable angina as the initial clinical presentation of CAD [15].

The purpose of this study is to explore the feasibility of a previously-described positron emission tomography (PET) imaging probe for the assessment of MMP-2/9 expression in mouse atherosclerotic plaques. Low-density lipoprotein receptor deficient mice (LDLR^-/-^ApoB^100/100^) used in this study develop inflamed atherosclerotic lesions throughout the aorta [16]. These lesions contain cell-rich, inflamed areas and acellular necrotic cores with occasional calcifications. Lipid rich, high cholesterol, Western-type diet further accelerates the development of atherosclerosis in this mouse model.

**Figure 1:**
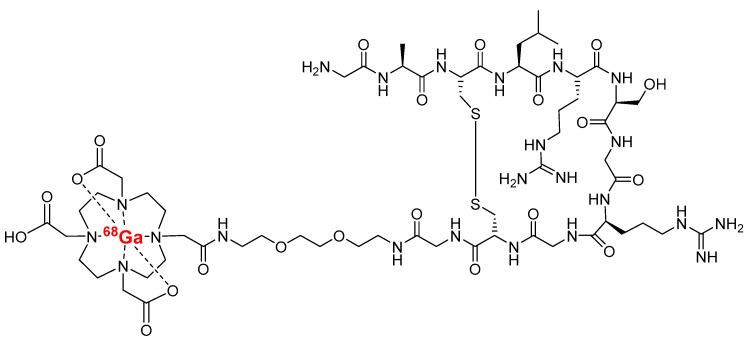
Structure of MMP-2/9 targeted [^68^Ga]Ga-DOTA-TCTP-1 tracer (Cys^3-10^; H-Gly-Ala-Cys-Leu-Arg-Ser-Gly-Arg-Gly-Cys-Gly-PEG(3)-DOTA-^68^Ga). DOTA, 1,4,7,10-tetraazacyclododecane-1,4,7,10-tetraacetic acid; PEG, polyethylene glycol.

The tracer is an 11-amino acid Cys^3^-Cys^10^ disulfide-bridged polyethylene glycol modified (PEGylated) peptide conjugated with 1,4,7,10-tetraazacyclododecane-1,4,7,10-tetraacetic acid (DOTA), which is labeled with gallium-68 (Figure 1). The peptide was discovered through biopanning (phage display) of living cells of a malignantly-transformed human cell line [17]. The tracer binds to activated MMP-2/9, and has already been evaluated for PET imaging in a mouse melanoma xenograft model [18] and a rat myocardial infarction model [19].

## 2. Results

### 2.1. Ex Vivo Biodistribution and Binding Specificity of MMP-2/9 Targeted Tracer

According to gamma counting of excised tissues at 60 min after i.v. injection, the tracer uptake in the whole aorta was at the same level in both atherosclerotic (0.43 ± 0.19 %ID/g) and control (0.52 ± 0.25 %ID/g, *p* = 0.61) mice (Table 1). In both mice strains, the highest radioactivity uptake was observed in kidneys and urine. The blood radioactivity concentration in atherosclerotic mice was 1.8-fold higher (1.3 ± 0.40 %ID/g, *p* = 0.017) than in the healthy controls (0.69 ± 0.21 %ID/g). Myocardial uptake of [^68^Ga]Ga-DOTA-TCTP-1 remained low in atherosclerotic mice (0.23 ± 0.083 %ID/g) and controls (0.16 ± 0.066 %ID/g, *p* = 0.54). Radioactivity was rapidly excreted through the kidneys (8.0 ± 2.9 %ID/g in atherosclerotic mice) to urine (440 ± 160 %ID/g).

The pre-treatment of mice with MMP-2/9 inhibitor decreased tracer uptake in atherosclerotic aorta by 55% (to 0.19 ± 0.013 %ID/g, *p* = 0.002), and in blood by 52% (to 0.60 ± 0.052 %ID/g, *p* = 0.030).

### 2.2. Autoradiography

**Figure 2:**
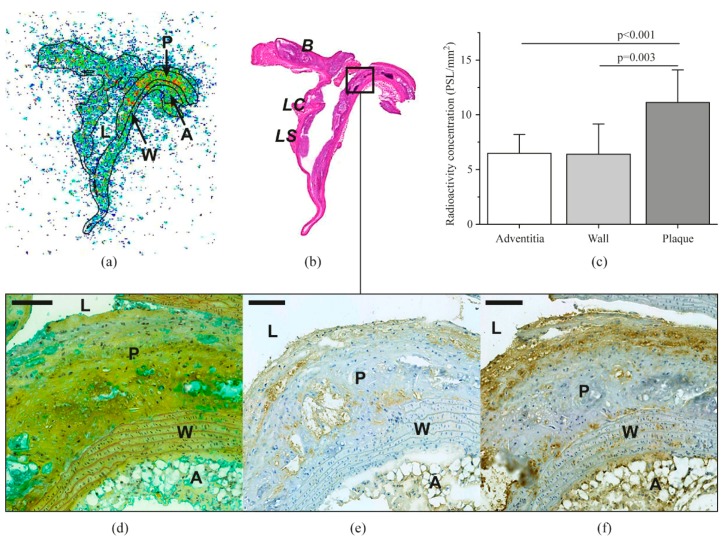
Distribution of [^68^Ga]Ga-DOTA-TCTP-1 in atherosclerotic mouse aorta as detected by digital autoradiography (**a**), and compared to anatomic landmarks after H&E staining (**b**). Panel (**c**) shows average tracer accumulation in the adventitia, normal vessel wall (wall), and atherosclerotic plaques (plaque). Micrographs show adjacent sections of an atherosclerotic plaque stained with Movat’s pentachrome (**d**), MMP-9 antibody (**e**), or Mac-3 antibody detecting macrophages (**f**). For details, see text. *A*, adventitia; *AA*, ascending aorta; *B*, brachiocephalic artery; *L*, lumen; *LC*, left carotid artery; *LS*, left subclavian artery; *P*, plaque; *W*, wall; *PSL*, photostimulated luminescence. Scale bars are 100 µm.

Representative autoradiographs of the aorta demonstrating uptake of the tracer in different regions (plaque, vessel wall, and adventitia) are shown in Figure 2. Quantitative autoradiography revealed that uptake of [^68^Ga]Ga-DOTA-TCTP-1 in atherosclerotic plaques (11 ± 3.0 PSL/mm^2^) of LDLR^-/-^ApoB^100/100^ mice was higher than in the normal vessel wall (6.4 ± 2.8 PSL/mm^2^; plaque-to-wall ratio: 1.8 ± 0.34, *p* = 0.0029) or adventitia (6.5 ± 1.7 PSL/mm^2^; plaque-to-adventitia ratio: 1.8 ± 0.49, *p* < 0.001).

### 2.3. In Vivo PET/CT

**Figure 3:**
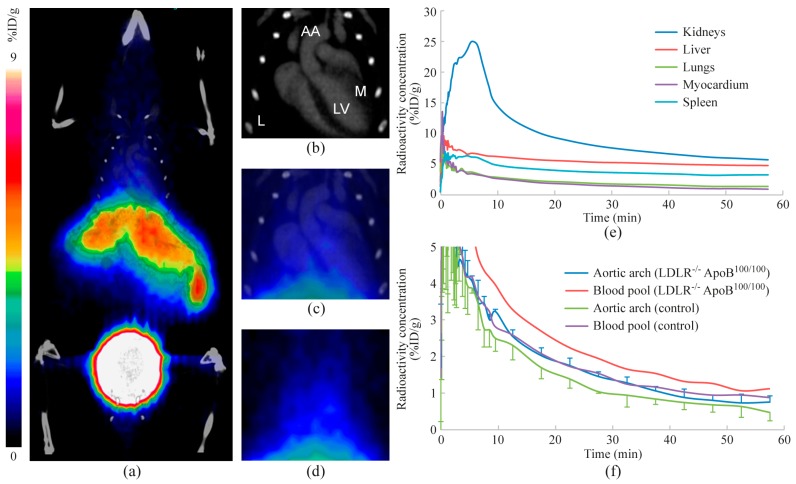
In vivo PET/CT imaging. Images represent radioactivity distribution at 30–60 min after injection. (**a**) A whole-body coronal PET/CT image, (**b**) contrast-enhanced CT image with anatomical landmarks (*AA*, aortic arch; *M*, myocardium; *L*, lung; *LV*, left ventricle), (**c**) combined PET/CT and (**d**) PET show a more detailed radioactivity distribution in the thoracic region. Mean time-activity curves represent selected tissues of three atherosclerotic mice (**e)**, as well as blood pool and aortic arch in both atherosclerotic (n = 3) and control mice (n = 3) (**f**).

Representative PET/CT images and time-activity curves are shown in Figure 3. Uptake in the blood pool remained higher than in the atherosclerotic aorta (1.1 ± 0.64 vs. 0.77 ± 0.16 %ID/g at 60 min after injection), and there was no focally increased uptake associated with the atherosclerotic lesions. The images also show rapid excretion of radioactivity through kidneys and variable uptake in the liver (4.7 ± 3.8 %ID/g at 60 min). Radioactivity remained low in the myocardium (0.81 ± 0.30 %ID/g at 60 min).

### 2.4. Histology and Immunohistochemistry

Atherosclerotic plaques in the aortas of LDLR^-/-^ApoB^100/100^ mice were mostly of the fibro-atheroma type, with a well-defined fibrous cap and macrophage infiltration visualized by Mac-3 staining (Figure 2f). Deposition of MMP-9 within the lesions was more diffuse, with only a few clearly positive cells, which were co-localized with Mac-3-positive macrophages (Figure 2e). The aortas of C57BL/6N control mice showed no evidence of atherosclerosis.

**Figure 4:**
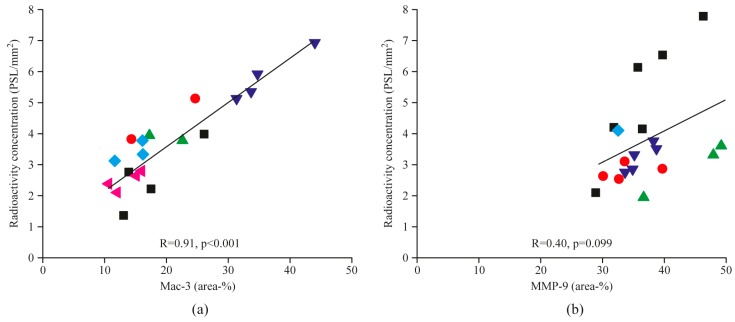
Scatter plots show correlations between areal percentages of Mac-3-positive macrophages (**a**), or MMP-9 (**b**), and corresponding [^68^Ga]Ga-DOTA-TCTP-1 uptake in the atherosclerotic plaques. Each symbol type represents plaques from the same animal. *R*, Pearson’s rank correlation coefficient; *PSL*, photostimulated luminescence.

The areal percentage of Mac-3-positive macrophages in plaque lesions closely correlated with uptake of the MMP-2/9 targeted tracer in the corresponding plaques (R = 0.91, *p* < 0.001, Figure 4a). However, MMP-9 staining (Figure 2e) did not show a statistically significant correlation (R = 0.40, *p* = 0.099) with tracer uptake (Figure 4b).

### 2.5. Zymography

Zymography revealed a high amount of activated MMP-9 (82 kDa) in the plasma of atherosclerotic mice, whereas active enzyme was not detectable in the aorta (Figure S1). We did, however, detect activated MMP-2 (64 kDa) in the aorta, although activity in the plasma appeared lower than that of MMP-9.

## 3. Discussion

We found an increased uptake of MMP-2/9-targeting [^68^Ga]Ga-DOTA-TCTP-1 in inflamed atherosclerotic plaques in LDLR^-/-^ApoB^100/100^ mice compared with normal vessel wall. Zymography confirmed the presence of activated MMP-2 in the aorta, and pre-treatment of atherosclerotic mice with MMP-2/9 inhibitor decreased tracer uptake in the aorta, indicating that the MMP-2/9-targeting [^68^Ga]Ga-DOTA-TCTP-1 detected gelatinase activation. Tracer uptake correlated closely with the quantity of macrophages, indicating that it may reflect inflammatory activity in atherosclerotic plaques. However, the blood radioactivity concentration remained higher than that of the aorta, and tracer uptake was not detectable in atherosclerotic lesions by in vivo PET/CT.

Our results are in line with those of previous studies where the uptake of broad spectrum MMP-targeting tracers correlated with intraplaque inflammation markers in apo-E deficient mice on a high fat diet [20] or which had undergone carotid artery ligation [21], and New Zealand White rabbits on a high fat diet [22]. Different MMP-targeting small molecule approaches [3] for single-photon emission computed tomography (SPECT) [20,21,22], PET [23,24], and optical/fluorescence imaging [25] have been successfully tested in cellular/tissue level assays [25], healthy mice [24], and various animal models of atherosclerosis [20,21,22]. Previous measurements of ex vivo uptake of broad spectrum MMP-targeting probes for fluorescence imaging [26] and SPECT [22] have shown 6–7-fold increases in atherosclerotic lesions in comparison with control vessels. Broad spectrum MMP-targeting SPECT probes have yielded promising results, with sufficient spatial resolution and target-to-background ratio to enable imaging in small-animal models of atherosclerosis [20,22]. Deguchi et al. [27] showed that a gelatinase-targeting activatable near-infrared fluorescence probe detected activation of MMP-2/9 in atherosclerotic aorta of apo-E deficient mice. In that study, MMP-2/9 activation was detectable by in vivo fluorescence molecular tomography. Our results are in line with those of their study, in showing the specific uptake of MMP-2/9 targeted [^68^Ga]Ga-DOTA-TCTP-1 in atherosclerotic lesions. However, in the present study the target-to-background ratio was insufficient for in vivo visualization by PET/CT. This may be partly explained by high residual activity in the blood.

As MMPs have a wide spectrum of physiological and pathophysiological roles, a question remains as to whether further development of MMP imaging probes should focus on wide spectrum MMP screening or on detecting more specific subpopulations and forms of various MMP enzymes. Currently, the development of gelatinase-selective probes has been difficult because of similarities in the enzyme structure of MMP-2 and MMP-9, although the affinity of auspicious probes can potentially be steered towards either one [24].

Although the exact role of MMP-9 in atherosclerosis remains controversial [20], MMP-9 activity has been found to be associated with carotid plaque in clinical trials where instability was visualized with ultrasound as a markedly irregular or ulcerated surface, or hypodense or heterogeneous structure [12], or when it was defined retrospectively according to a thin fibrous cap and/or recent intraplaque hemorrhage on histology [13], as well as being indicated by previous stroke or peripheral vascular disease [13]. Circulating levels of MMP-2/9 are also associated with the development of an acute myocardial infarction rather than stable angina, as the initial clinical presentation of coronary artery disease [15]. Still, a further understanding of the pathophysiological roles of each MMP subtype in each of its different forms, and their exact time course in relation to plaque size and the development of vulnerability, is warranted to facilitate tracer development and the establishment of a clinical imaging-based plaque vulnerability screening protocol. Particularly, our results suggest, that the affinity of gelatinase imaging probes should be steered towards activated MMP-2, to reduce the interference of circulating enzymes on the target visualization in vivo.

## 4. Materials and Methods

### 4.1. Animal Model and Other Materials

Sixteen (eleven males, five females) low-density lipoprotein receptor deficient mice expressing only apolipoprotein B100 (LDLR^-/-^ApoB^100/100^, Jackson Laboratory, Bar Harbor, ME, USA, strain #003000) were used. Mice were kept on a Western-type diet for 4.4 months starting at the age of 3.5 months. Forty-two percent of their calorific intake consisted of fat and 0.2% cholesterol (TD 88137, Harlan Teklad, Harlan Laboratories, Madison, WI, USA). Nine C57BL/6N mice on a regular chow diet (five males, four females) were used as controls. The mean age and weight of the studied mice at the time of tracer injection were 8.2 ± 0.70 months and 38 ± 8.0 g for the LDLR^-/-^ApoB^100/100^ mice, and 3.9 ± 0.86 months and 31 ± 5.8 g for the controls. During the studies, mice were housed under standard conditions (light and humidity) with ad libitum access to water and food. The study protocol was approved by the National Animal Experiment Board in Finland and the Regional State Administrative Agency for Southern Finland, and carried out in compliance with the relevant European Union directives. The high-performance liquid chromatography (HPLC) system was a LaChrom instrument (Hitachi; Merck Darmstadt, Germany), including a pump L7100, an ultraviolet (UV) detector L-7400, an interface D-7000 and a computerized data acquisition and processing software. The reversed-phase HPLC column (μBondapak 10 μm C18, 7.8 × 300 mm, pore size 125 Å) was purchased from Waters Corporation, Milford, MA, USA. The online radioisotope detector (Radiomatic 150 TR, Flow Scintillation Analyzer) was from Packard, Meriden, CT, USA. The precursor compound DOTA-TCTP-1 (Figure 1) was obtained as a custom synthesis from Peptide Specialty Laboratories GmbH (Heidelberg, Germany).

### 4.2. Radiochemistry

[^68^Ga]Ga-DOTA-TCTP-1 was prepared as previously described [17]. The radionuclide ^68^Ga was obtained from a ^68^Ge/^68^Ga-generator (Eckert & Ziegler Isotope Products, Burbank, USA) in the form of [^68^Ga]GaCl_3_. Radiolabeling was performed by adding DOTA-TCTP-1 (14–30 nmol) into a solution of [^68^Ga]GaCl_3_ (500 µL) buffered with sodium acetate (219 µmol) at pH 4.0. The reaction mixture was incubated at 95 °C for 10–25 min and then cooled down to room temperature. The pH of the end product was adjusted to 6−7 with 1 M NaOH. The radiochemical yield was quantitative and there was no need to perform purification. Radiochemical purity was measured by HPLC with both radioactivity detection and ultraviolet detection (215 nm). The flow rate was 6 mL/min. Solvent A was 50 mM phosphoric acid, solvent B was 50 mM ammonium acetate/acetonitrile (1:1 by volume) and solvent C was 50 mM ammonium acetate. The gradient was programed with a composition of A/B/C as follow: 0–3 min 0/15/85, 3–4 min 0/30/70, 4–5 min 0/60/40, 5–9 min 0/70/30, and 9–13 min 100/0/0. The molar activity was 14.7 ± 7.1 GBq/µmol (n = 12), and radiochemical purity 97.9 ± 1.0% (n = 12).

### 4.3. Ex Vivo Biodistribution

Isoflurane anesthetized mice were intravenously (i.v.) injected with 18 ± 2.7 MBq (in 50–100 µL) of [^68^Ga]Ga-DOTA-TCTP-1 via the tail vein. At 60 min after tracer injection, a blood sample was obtained by cardiac puncture and animals were killed by cervical dislocation. Various tissues were excised, weighed, and measured for radioactivity on a gamma counter (Triathler 3”, Hidex, Turku, Finland). Radioactivity values were normalized by the injected radioactivity dose decay with a delta time between injection and measurement, animal weight, and the weight of tissue. Results are expressed as the percentage of injected radioactivity dose per gram of tissue (%ID/g).

To assess the specificity of [^68^Ga]Ga-DOTA-TCTP-1 accumulation in various tissues, four (three males, one female) LDLR^-/-^ApoB^100/100^ mice were i.v. injected with 1.4 µmol/kg (approximately 20 µmol/L of blood volume, IC_50_ = 10 µmol/L) of specific MMP-2/9 inhibitor [H-Cys^1^-Thr-Thr-His-Trp-Gly-Phe-Thr-Leu-Cys^10^-OH (cyclic: 1→10)] (product number: 444251, Merck KGaA, Darmstadt, Germany) 5 min prior to the administration of [^68^Ga]Ga-DOTA-TCTP-1.

### 4.4. Autoradiography

The aortas were excised, frozen in cooled isopentane, and sliced into serial longitudinal cryosections of 8 and 20 µm; then the accumulation of [^68^Ga]Ga-DOTA-TCTP-1 in different areas of the aorta was analyzed by autoradiography. Air-dried sections were opposed to an imaging plate (Fuji Imaging Plate BAS-TR2025, Fuji Photo Film Co., Tokyo, Japan). After more than two radionuclide half-lives, the plates were scanned with a Fuji BAS-5000 analyzer (Fuji, Tokyo, Japan; internal resolution of 25 μm). Tracer accumulation was measured as counts per area (photostimulated luminescence per square millimeter, PSL/mm^2^) using TINA software v. 2.1 (Raytest Isotopenmessgeräte, GmbH, Straubenhardt, Germany). After careful co-registration of autoradiography and histological images, regions of interest (ROIs) defining plaque lesions, vessel wall, and adventitia were created. Background area count densities were subtracted from the image data. Uptake in the autoradiographs was normalized using a mathematical algorithm taking into account the radionuclide decay, injected radioactivity dose, time from injection to imaging plate exposure, and exposure time. Accumulation was measured primarily from 20 µm sections, with a comparison with immunohistochemistry being performed using the adjacent 8 µm sections.

### 4.5. In Vivo PET/CT

A subset of mice (three atherosclerotic and three healthy controls) were imaged using a small-animal PET/computed tomography (CT) scanner (Inveon Multimodality; Siemens Medical Solutions, Knoxville, TN, USA). The mice were anesthetized using 1.5% isoflurane, and their temperature was maintained using a heating pad throughout the imaging. Mice were i.v. injected with 11 ± 0.86 MBq (in 50–100 µL) of [^68^Ga]Ga-DOTA-TCTP-1 via the tail vein. PET data were acquired in a list-mode for 60 min, starting from the time of injection of [^68^Ga]Ga-DOTA-TCTP-1. The images were reconstructed using an ordered-subset expectation maximization 2D algorithm with four iterations into 30 × 3 s, 9 × 10 s, 4 × 30 s, 5 × 60 s, and 10 × 300 s time frames. Immediately after the PET scan, 100 µL of intravascular iodinated contrast agent eXIATM160XL (Binitio Biomedical Inc., Ottawa, ON, Canada) was injected and high-resolution CT was acquired (80 kV, 500 μA). CT images were reconstructed using a Feldkamp-based algorithm, and images were analyzed using Carimas v.2.6 software (Turku PET Centre, Turku, Finland). ROIs were drawn in the aortic arch, blood pool (inside the LV cavity), and several relevant organs, according to the high-resolution CT image. Results are reported as the percentage of injected dose per gram (%ID/g) as a function of the time after injection, i.e., as time-activity curves.

### 4.6. Histology and Immunohistochemistry

After the autoradiography, serial aortal cryosections were stained with hematoxylin-eosin (H&E) or Movat’s pentachrome for general histology. The percentages of positive immunostaining in each atherosclerotic lesion were plotted against the normalized tracer accumulation measured by digital autoradiography. Photomicrographs of the immunostained sections were captured with a Pannoramic 250 Flash digital slide scanner (3DHISTECH Ltd., Budapest, Hungary), stain contrast was digitally enhanced, and the densities of the positive immunostainings in separate plaques were analyzed with Image-J software v. 1.46 (National Institutes of Health, Bethesda, MD, USA). For enumeration of activated macrophages and MMP-9 detecting immunohistochemistry, the cryosections were incubated with rat anti-mouse Mac-3 antibody at a 1:5000 dilution (product number: 550292, Clone M3/84, BD Pharmingen, Franklin Lakes, NJ, USA), or rabbit polyclonal anti-MMP-9 antibody at a 1:1000 dilution (ab38898, Abcam, Cambridge, UK), with the horseradish peroxidase-conjugated secondary antibody being finally visualized using 3,3-diaminobenzidine (DAB).

### 4.7. Zymography

Fresh aorta and plasma samples of five LDLR^-/-^ApoB^100/100^ mice were homogenized (aortas) and diluted into zymogram sample buffer (BioRad, Hercules, CA, USA), and an equal volume was added to each lane of the zymogram gels (10% gelatin; BioRad). The gels were run, renatured, and developed using BioRad zymogram buffers, according to the manufacturer’s instructions. Gels were stained using Coomassie Brilliant Blue, and destained using a solution containing methanol and acetic acid.

### 4.8. Statistical Analysis

All data are expressed as mean ± SD to two significant figures. Statistical analysis was performed with SPSS v. 24 (IBM, NY, USA). For comparison of different tissues within the same subject, Student’s t-test for paired data was applied. An independent-samples Mann-Whitney U test was used for comparisons between two study groups. The Pearson 2-tailed product-moment correlation coefficient was used for assessing correlations between two continuous variables. P values less than 0.05 were considered statistically significant.

## 5. Conclusions

The MMP-2/9-targeting [^68^Ga]Ga-DOTA-TCTP-1 shows uptake in inflamed mouse atherosclerotic lesions, but because of a low target-to-background ratio, the tracer did not permit in vivo imaging of MMP-2/9 activation in the vessel wall. Our results suggest, that the affinity of gelatinase imaging probes should be steered towards activated MMP-2, to reduce the interference of circulating enzymes on the target visualization in vivo.

## Figures and Tables

**Table 1:** Ex vivo biodistribution of MMP-2/9 targeted [^68^Ga]Ga-DOTA-TCTP-1 in mice at 60 min after intravenous injection.

	Atherosclerotic LDLR^-/-^ ApoB^100/100^ (n = 9)	Control C57BL/6N (n = 9)	*P* Atherosclerotic vs. Control	Inhibitor Pre-Treated Atherosclerotic (n = 4)	*P* Atherosclerotic vs. Inhibitor Pre-Treated
Aorta	0.43 ± 0.19	0.52 ± 0.25	0.61	0.19 ± 0.013	0.0020
Blood	1.3 ± 0.40	0.69 ± 0.21	0.017	0.60 ± 0.051	0.030
Bone	0.53 ± 0.37	0.63 ± 0.50	0.10	0.27 ± 0.080	0.030
Heart	0.23 ± 0.083	0.16 ± 0.066	0.54	0.12 ± 0.054	0.0050
Intestine	0.59 ± 0.22	0.43 ± 0.18	0.16	0.25 ± 0.15	0.093
Kidneys	8.0 ± 2.9	8.9 ± 3.6	0.96	7.3 ± 3.0	0.354
Liver	1.8 ± 0.48	3.2 ± 1.6	0.081	3.6 ± 2.4	0.0040
Lungs	2.8 ± 1.6	1.3 ± 0.74	0.038	2.0 ± 1.7	0.0020
Lymph node	0.49 ± 0.15	0.48 ± 0.19	0.88	0.30 ± 0.089	0.22
Pancreas	0.35 ± 0.17	0.17 ± 0.056	0.015	0.15 ± 0.062	0.019
Plasma	2.6 ± 1.4	1.5 ± 1.0	0.029	1.1 ± 0.10	0.048
Salivary gland	0.38 ± 0.15	0.23 ± 0.070	0.065	0.19 ± 0.033	0.17
Skeletal muscle	0.19 ± 0.077	0.17 ± 0.13	0.14	0.10 ± 0.027	0.029
Spleen	1.9 ± 1.6	1.7 ± 1.3	0.54	2.5 ± 1.7	0.018
Thymus	0.35 ± 0.29	0.23 ± 0.070	0.57	0.12 ± 0.033	0.020
Urine	440 ± 160	320 ± 150	0.28	210 ± 39	0.52
WAT	0.17 ± 0.21	0.17 ± 0.11	0.38	0.24 ± 0.21	0.0060

The results are expressed as percentage of injected radioactivity dose per gram of tissue (%ID/g, mean ± SD). *P* values are from independent-samples Mann-Whitney U tests. WAT, white adipose tissue.

## Supplementary Materials

The following are available online, Figure S1: Zymographs of atherosclerotic mouse aortas (a) and plasma (b).
