# Supplementary material for: Evaluation of [68Ga]Ga-DOTA-TCTP-1 for the Detection of Metalloproteinase 2/9 Expression in Mouse Atherosclerotic Plaques
Source: Molecules. 2018 Dec 1;23(12):3168. doi: 10.3390/molecules23123168 (PMC6321344; doi:10.3390/molecules23123168)
Supplement: Supplementary file 1 [file molecules-23-03168-s001.pdf]

molecules

Supplementary material

Evaluation of [^{68}Ga]Ga-DOTA-TCTP-1 for the detection of metalloproteinase 2/9 expression in mouse atherosclerotic plaques

Max Kiugel[1], Sanna Hellberg[1], Meeri Käkelä[1], Heidi Liljenbäck[1,2], Tiina Saanijoki[1], Xiang-Guo Li[3], Johanna Tuomela[3], Juhani Knuuti[1,5], Antti Saraste[1,5,6,7], Anne Roivainen[1,2,5,*]

1 Turku PET Centre, University of Turku, Turku, Finland; max.kiugel@utu.fi (M.K.); sanna.hellberg@utu.fi (S.H.); meeri.kakela@utu.fi (M.K.); halilj@utu.fi (H.L.); tiina.saanijoki@utu.fi (T.S.); juhani.knuuti@utu.fi (J.K.); antti.saraste@utu.fi (A.S.); anne.roivainen@utu.fi (A.R.)
2 Turku Center for Disease Modeling, University of Turku, Turku, Finland
3 Turku PET Centre, Åbo Akademi University, Turku, Finland; xiali@utu.fi (X-G.L)
4 Department of Cell Biology and Anatomy, University of Turku, Turku, Finland; johanna.tuomela@utu.fi (J.T.)
5 Turku PET Centre, Turku University Hospital, Turku, Finland
6 Heart Center, Turku University Hospital, Turku, Finland
7 Institute of Clinical Medicine, University of Turku, Turku, Finland
* Correspondence: anne.roivainen@utu.fi; Tel.: +358-2-3132862

Received: 23 November 2018; Accepted: 29 November 2018; Published: date

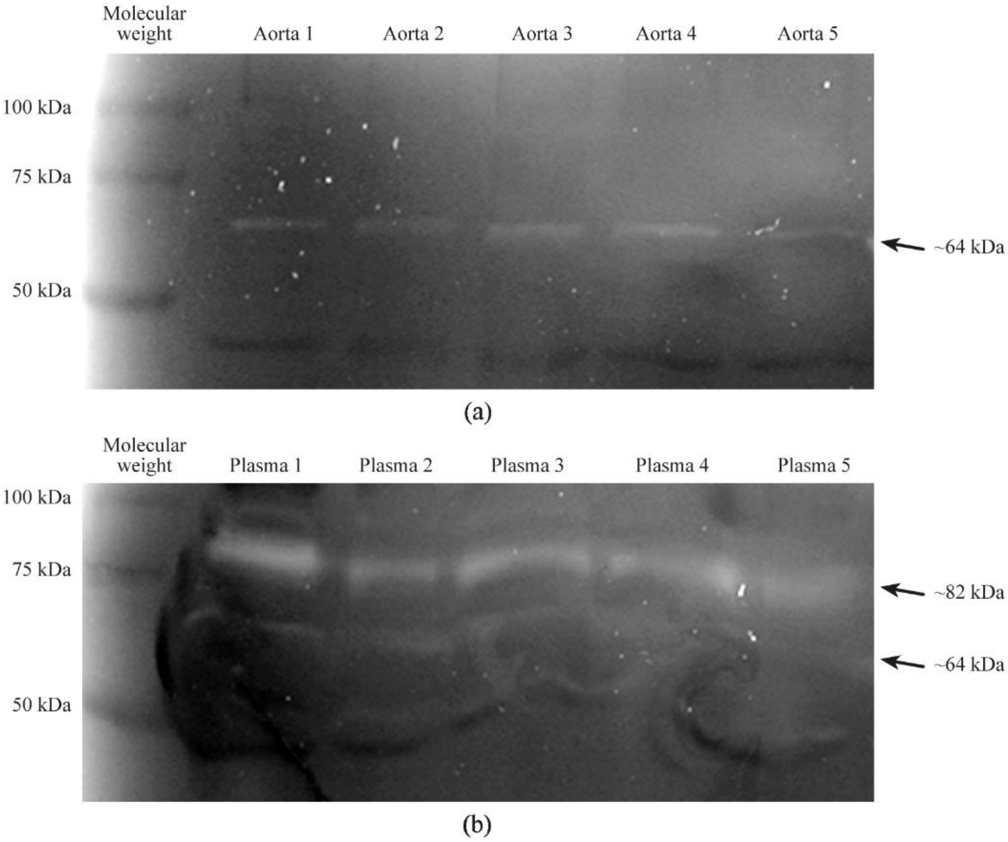

Supplementary Figure 1. Zymographs of atherosclerotic mouse aortas (a) and plasma (b). The molecular weights of activated MMP-2 and MMP-9 are 64 kDa and 82 kDa, respectively. Dark bands represent molecular weight markers, and white bands are positive signals where enzymatic activity has degraded the gel structure.
